# The Use of Wearable Devices to Measure Sedentary Behavior during COVID-19: Systematic Review and Future Recommendations

**DOI:** 10.3390/s23239449

**Published:** 2023-11-27

**Authors:** Yehuda Weizman, Adin Ming Tan, Franz Konstantin Fuss

**Affiliations:** 1 Chair of Biomechanics, Faculty of Engineering Science, University of Bayreuth, D-95447 Bayreuth, Germany; franz.konstantin.fuss@ipa.fraunhofer.de; 2 Department of Health and Medical Sciences, School of Health Sciences, Hawthorn Campus, Swinburne University of Technology, Melbourne 3122, Australia; adintan@gmail.com; 3 Division of Biomechatronics, Fraunhofer Institute for Manufacturing Engineering and Automation IPA, D-95447 Bayreuth, Germany

**Keywords:** sedentary behavior, physical activity, COVID-19, pandemic, wearable devices, inertial measurement unit (IMU)

## Abstract

The SARS-CoV-2 pandemic resulted in approximately 7 million deaths and impacted 767 million individuals globally, primarily through infections. Acknowledging the impactful influence of sedentary behaviors, particularly exacerbated by COVID-19 restrictions, a substantial body of research has emerged, utilizing wearable sensor technologies to assess these behaviors. This comprehensive review aims to establish a framework encompassing recent studies concerning wearable sensor applications to measure sedentary behavior parameters during the COVID-19 pandemic, spanning December 2019 to December 2022. After examining 582 articles, 7 were selected for inclusion. While most studies displayed effective reporting standards and adept use of wearable device data for their specific research aims, our inquiry revealed deficiencies in apparatus accuracy documentation and study methodology harmonization. Despite methodological variations, diverse metrics, and the absence of thorough device accuracy assessments, integrating wearables within the pandemic context offers a promising avenue for objective measurements and strategies against sedentary behaviors.

## 1. Introduction

On 30 January 2020, the World Health Organization (WHO) officially designated the coronavirus outbreak as a global public health emergency [1]. Across a span of three years, the SARS-CoV-2 pandemic led to the death of nearly 7 million individuals and impacted over 767 million people with infections on a global scale [2].

During the COVID-19 pandemic and its resulting lockdown measures, research [3] showed a multitude of significant factors that emerged as primary contributors to obesity, encompassing aspects such as insufficient physical activity, sedentary behaviors, and compromised sleep quality. 

A sedentary lifestyle, a pressing public health concern, refers to a way of living that involves minimal physical movement and low energy expenditure, often associated with walking, sitting, or reclining postures [4]. This lifestyle can have significant consequences on our overall health and well-being, leading to a range of physical and mental health issues. An increasing body of epidemiological evidence has established a connection between sedentary behavior and a range of health risks, including an elevated likelihood of developing type 2 diabetes, metabolic syndrome, cancer, and obesity, as well as increased mortality rates associated with all causes and cardiovascular disease [5,6,7,8,9,10]. Recent research has contributed significant insights into the impact of the pandemic on physical activity and sedentary behavior, and certain studies have attempted to explore the factors associated with these changes in behavior [11,12,13,14].

Hence, the measurement of a sedentary lifestyle particularly during the COVID-19 pandemic has been important for several specific reasons such as the impact of lockdowns, health consequences, risk identification, and public health preparedness for future crises. 

In recent years, many people have incorporated personal wearable devices into their daily lives. The integration of noninvasive wearable motion sensors has become a fundamental clinical tool within the healthcare industry, establishing itself as a common practice [15]. In particular, inertial measurement units (IMUs) have enabled the accurate estimation of kinematic parameters such as body position, acceleration, and speed, demonstrating remarkable precision [16]. Fitbit (Fitbit, San Francisco, CA, USA) and activPAL (Physical Activity Technologies, Glasgow, Scotland) are two widely utilized wearable devices in scientific research that provide objective measurements of sedentary behavior. Fitbit is designed to be worn on the wrist and offers continuous monitoring, while activPAL was designed to be worn on the thigh to categorize free-living activity into sitting, standing, and stepping behaviors. While the utilization of these devices offers a potential advantage in terms of objective and measurable outcomes compared to conventional subjective methods, there are still challenges in standardization procedures and limited comparability among studies [17]. 

Given the impact of the COVID-19 pandemic on physical activity and sedentary behaviors, coupled with the wide prevalence of use of these devices, they have emerged as essential tools in COVID-19 research. These devices played a key role in collecting real-time, objective, and large-scale data on physical activity, enabling remote monitoring and telehealth services. Recent studies have demonstrated the utility of wearable devices in monitoring sedentary life during COVID-19 [18,19,20,21,22,23,24]. In their study, Leon et al. [22] utilized commercialized ambient (vision) and wearable sensors within a controlled environment to achieve real-time recognition. The study focuses on four human postures, standing, sitting, bending, lying down, and walking activity, while also quantifying energy expenditure. The results obtained showcased the platform’s automatic energy expenditure quantification capabilities using both individual wearable and vision-sensing technologies, as well as a combined version, achieving average accuracies greater than 93%. In another study [21] by MAIR et al., the group developed JitaBug, a personalized Just-in-Time Adaptive Intervention (JITAI) smartphone application. The study aimed for usage in a real-world environment to assist older adults in increasing or maintaining their physical activity levels. Furthermore, this study assesses the feasibility of executing an efficacy trial for JitaBug while also scrutinizing its acceptability in a real-world context among older adults. The initial findings suggest that employing JITAI is favorably received for aiding physical activity among older adults residing in the community; however, improvements to the technical facets of the JitaBug app are needed before progressing to efficacy trials, aiming to amplify user-friendliness, engagement, and overall user satisfaction. 

Recently, multiple experts in different disciplines across public health, epidemiology, and infectious diseases have formulated recommendations, informed by existing references, to guide the management of future global pandemics [2,25,26,27]. In this post-mortem period, to the author’s knowledge, not much attention has been paid to sedentary behavior and the importance of maintaining consistent levels of physical activity during a pandemic. Developing evidence-based protocols for maintaining physical activity levels during such periods is essential for safeguarding individual health and well-being.

This systematic review aims to provide an updated framework of the current research on the use of wearable sensors to measure sedentary behavior parameters during the coronavirus pandemic. In addition, the authors will provide their perspective on potential future application recommendations in the field. 

## 2. Methods

### 2.1. Search Strategy

A systematic literature search was performed to identify the most relevant studies according to the Preferred Reporting Items for Systematic Reviews and Meta-Analyses (PRISMA) checklist [28]. The following electronic databases were searched: Cochrane Library, IEEE Xplore, and PubMed including MEDLINE database of references and abstracts on life sciences and biomedical topics to identify articles published during a 3-year period, since the official COVID-19 pandemic began, from December 2019 to December 2022. The specific search keywords and Boolean combinations used were ((wearable*) OR (Sensor*) OR (Wearable device) OR (wearable sensor) OR (body worn) OR (device*) OR (tracker*) OR (IMU) OR (Fitbit)) AND ((Sedentary*) OR (sedentary behaviour) OR (sedentary time) OR (sedentary lifestyle) OR (physical activity) OR (exercise) OR (walk*) OR (sit*)) AND ((COVID*) OR (SARS-CoV-2)).

### 2.2. Study Selection Strategy

After removing duplicate manuscripts, two reviewers (Y.W. and A.M.T) independently screened the title, abstract, and keywords of the records identified through database searching. We used RAYYAN [29], an online systematic review tool, for the literature management.

The inclusion criteria for articles were as follows:

(1) Articles published in English.
(2) Full original research articles published in peer-reviewed scientific journals.
(3) Studies involving healthy adult human participants, aged between 18 and 75 years, with no pre-existing health conditions.
(4) Studies focused on measuring sedentary behavior related to COVID-19 using characteristics derived from wearable devices or body-worn sensors.
(5) Wearable devices need to be small, portable, easy to use, and unobtrusive for the desired analysis.

To streamline the study selection and classification of retrieved papers, we defined the following exclusion criteria:

(1) Studies that used animal models.
(2) Conference papers.
(3) Studies unrelated to sedentary behavior and COVID-19.
(4) Adults with chronic lifestyle illnesses were excluded, as they might have fitness plans, dietician recommendations leading to physical mobility issues, or other physiological considerations, mental health issues, or health-related motivations.
(5) Studies that did not primarily focus on wearable device analysis.
(6) Studies that utilized invasive wearables.
(7) Studies that employed wearables solely to track working status.
(8) Studies that only proposed protocols without presenting results.
(9) Studies primarily focused on virus spread prevention and social distancing.
(10) Studies concentrating on robotics were also excluded.

### 2.3. Data Extraction

Two reviewers (Y.W. and A.M.T.) thoroughly analyzed the articles, and the relevant information was extracted and organized into two tables. Table 1, titled “Study characteristics”, comprises details such as the aim of the study, characteristics of the recruited population, selection criteria, and participants’ demographics. Table 2, named “Study parameters and outcome measures”, provides information on the sensor type and specifications used in each study, the location on the body where the sensor was placed, the calculated physical activity parameters, the sensor assessment protocol employed, the environment (e.g., controlled or free-living) in which the study took place, and the main findings derived from the research.

### 2.4. Methodological Quality 

The quality evaluation of each included article was performed using a custom quality assessment worksheet (Table 3) based on the reviews conducted by Benson et al. and Campos et al. [30,31]. The table comprises 12 items that are divided into four sub-scales: reporting, external validity, internal validity (bias), and power analysis. Two authors, namely Y.W. and A.M.T, independently assessed the quality of each study included in this systematic review. For each item, three possible answers were provided: “Yes” or “No”. In case of any discrepancies in scoring between the authors, they discussed the disagreements until they reached a consensus.

## 3. Results

### 3.1. Search Results 

**Figure 1:**
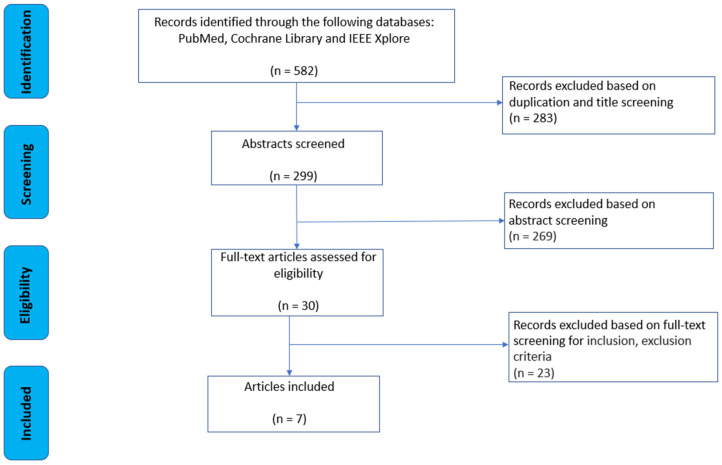
Strategy of literature review process flow chart.

Figure 1 represents the study selection process conducted across three databases. The systematic review initially identified 582 studies, and after removing duplicates, 299 relevant studies remained. These manuscripts underwent further detailed full-text analysis based on the inclusion/exclusion criteria, resulting in 30 publications for closer examination. Finally, seven final papers [18,19,20,21,22,23,24] met the criteria and were included in this systematic review. The primary reason for excluding most studies in the final phase was the presence of participants with mental or health backgrounds that did not align with the study’s focus.

### 3.2. Study Characteristics and Reported Limitations

Table 1 provides an overview of the study and participant characteristics of the seven selected studies, which utilized different commercially available wearable devices to assess various physical activity measures for different objectives. One study [18] focused on evaluating responses to digital message interventions, while Gilley et al. [19] identified risk factors associated with COVID-19 positivity in a population of college students before the vaccine’s release. Massar at al. [20] examined the evolution of physical activity behaviors during reopening, and Mair et al. [21] described the development of JitaBug, a personalized smartphone-delivered intervention application. Leone et al. [22], Capodilupo et al. [23], and Ong et al., 2021 [24] studied changes in sleep and physical activity behaviors during COVID-19 physical distancing restrictions. The total sample size across the studies ranged from 11 to 5436, encompassing different population characteristics such as young adults, college students [18,19], aging adults [22], and mixed-gender groups. Regarding the selection standards, five studies reported their inclusion criteria [18,19,21,23,24], while three studies reported their exclusion criteria [18,19,21].

In Capodilupo et al. [23], limitations include potential bias in the US-based WHOOP user demographic, limited global generalizability and adherence to physical distancing, reliance on an estimated timeline for period distinction, and concerns about inflated significant findings due to multiple testing. In Leone et al.’s [22] study, limitations stem from a small sample of aging subjects, challenging statistical robustness, difficulties in evaluating subjects with mobility disorders, and using a methodology misaligned with gold-standard energy expenditure measurements. Restricted monitoring due to sensor device operating ranges further limits the study’s representation of real-world activities. Mair et al.’s [21] study relies on Fitbit trackers, which are potentially less valid compared to research-grade accelerometers (e.g., ActiGraph), for activity intensity assessment, while muscle-strengthening activities are underrepresented. A lack of contextual data utilization and a predominantly well-educated demographic raise generalizability concerns. Gilley et al.’s [19] study faces limitations due to the overrepresentation of Asian students, gender imbalance, and potential data attrition. The reliance on self-reports for COVID-19 diagnosis and limited validation pose challenges. However, the study demonstrates the feasibility of longitudinal research during a pandemic using mobile health technology. Massar et al.’s [20] study uses a convenience sample with limited generalizability, skewed gender ratios, and a lack of pre-lockdown data. Sample size estimation challenges are addressed by combining objective tracking with self-reported data to minimize reporting biases. Hojjatinia’s [18] study deals with limitations including temporary changes in living arrangements and employment status. The use of dynamical system modeling is effective, but reinforcement-learning algorithms are impractical due to the data size. Instead, system identification methods are used for efficient analysis. Ong’s [24] study relies on consumer-grade sleep tracking and lacks data on sleep disorders. The ongoing nature of the study and potential future lockdowns limit generalizability, while concerns exist about tracking short daytime naps and the influence of government financial support on sleep patterns in socioeconomically distressed populations.

### 3.3. Study Parameters and Outcome Measures 

Table 2 presents a concise overview of the parameters and outcome measures extracted from the selected studies. In order to gather the necessary data, all of the selected studies utilized one or more wearable devices that were affixed to the participants’ bodies to capture information regarding their physical activity.

#### 3.3.1. Sensor Type and Body Location 

All the studies included in the analysis provided specific information regarding the brands of physical activity trackers they employed. Among the selected studies, three [18,21,22] explicitly mentioned their sampling frequency, which varied between 30 and 100 Hz. The Fitbit wristband (Fitbit, Inc., San Francisco, CA, USA) was utilized in four studies [18,19,21,24], while the ActiGraph (ActiGraph, Pensacola, FL, USA) was used in two studies [18,21]. Additionally, one study [22] utilized the Shimmer tracker (Shimmer, Dublin, Ireland), and another study [23] utilized the WHOOP strap (WHOOP, Inc., Boston, MA, USA). Regarding the placement of the physical activity trackers on the body, all studies reported varying locations. Specifically, the trackers were positioned around the wrist or finger [18,19,20,21,23,24], on the thigh [18], and on the chest [22].

#### 3.3.2. Key Sedentary Parameters and Assessment Procedures 

The physical activity data obtained from the body-worn trackers underwent processing to derive variables that characterized various aspects during different assessment procedures. All studies [18,19,20,21,22,23,24] focused on investigating the total number of steps taken per minute, per day, throughout the entire duration of the study and walking poses. Additionally, heart rate data were collected and analyzed in four studies [18,19,23,24] to assess variability, resting heart rate, and sleep-related parameters such as bed and lying time. Five studies [19,20,22,23,24] reported data on sleep-related variables. One study [22] conducted its assessments in a controlled environment, which included tasks such as standing, sitting, bending, and lying down, with each task lasting between 30 and 90 s. On the other hand, the remaining six studies [18,19,20,21,23,24] collected physical activity data in a real-world, day-to-day setting, capturing information over different periods of time. Hojjatinia et al. [18] conducted their study in two stages: a screening phase lasting 7 days, followed by a 6-month intervention phase. Other studies by Gilley et al. [19], Massar et al. [20], and Mair et al. [21] collected data over durations of three months, eight weeks, and eight consecutive days, respectively.

### 3.4. Methodological Quality 

The results of the quality assessment, as depicted in Table 3, provide valuable insights into the evaluated research studies. Upon thorough analysis, it is evident that these studies generally demonstrate a fair level of proficiency in explaining their current level of knowledge, research objectives, and significant findings. However, it is important to highlight certain areas that require improvement and further clarification.

Specifically, there are notable deficiencies in the description of participant characteristics in terms of mean age and health status across certain studies [19,20,21,22]. This lack of clarity hinders a comprehensive understanding of the demographic composition of the study populations, which could potentially impact the generalizability and applicability of the findings. Furthermore, a significant number of studies [19,20,21,22,23,24] failed to provide any information regarding their inclusion or exclusion criteria. This omission limits the transparency and reproducibility of their methodologies, making it difficult to assess the extent to which the selected participants represent the target population.

On a positive note, all studies effectively described their findings and main outcomes, enabling readers to grasp the essence of their research endeavours. It is worth noting that the participants in these studies were drawn from a diverse population, encompassing both young and aging adults, with an age range spanning from 18 to 73 years. The test settings and conditions employed in the majority of the studies were representative of real-life environments, where continuous movement data were collected using sensors. However, it is important to highlight that Leon’s study [22] deviated from this approach by conducting experiments in a controlled environment. This distinction should be taken into consideration when assessing the generalizability of the results.

In terms of statistical analyses, all studies adequately defined the utilized tests and outcome measures, which contributes to the rigor and validity of their findings. However, it is worth mentioning that none of the studies provided justification for their chosen sample sizes or a description of any conducted power analysis. The absence of such details raises questions about the adequacy of the sample sizes employed and the statistical power of the studies. Consequently, further elaboration on these aspects is necessary to ensure the reliability and robustness of the reported findings.

## 4. Discussion

**Figure 2:**
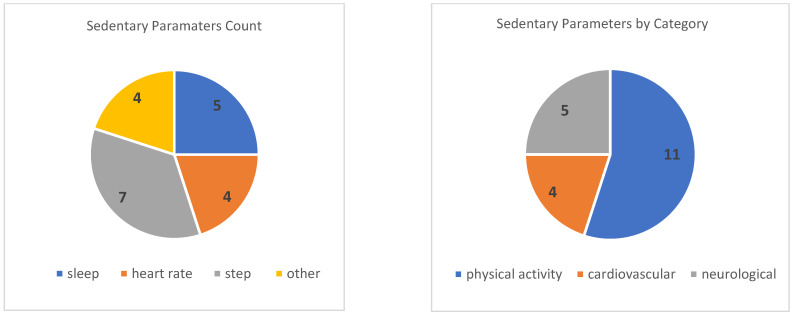
(**left**): reported sedentary parameters count; (**right**): number of sedentary parameters by category.

The COVID-19 pandemic has had a significant impact on global health, resulting in the implementation of widespread public health measures to control its transmission [32]. This systematic review aims to provide an analysis of the utilization of wearable devices in assessing sedentary lifestyle parameters between December 2019 and December 2022 of the COVID-19 outbreak and propose potential future directions based on the existing research findings. Seven studies [18,19,20,21,22,23,24] were identified, employing diverse study protocols and settings, including laboratory and real-world environments. Table 2 presents a summary of the included studies, all of which demonstrated the effectiveness of wearable sensor-based devices in achieving their specific objectives. In addition, Table 4, adapted from Prill et al. [33], outlines an instant view of all key study and sensor characteristics, and Figure 2 illustrates sedentary parameters count and number of sedentary parameters by category, highlighting the top measured parameters count by category.

Multiple studies addressed various aspects of health interventions during the COVID-19 pandemic. One study [18] assessed digital message intervention impacts, while another [19] identified COVID-19 risk factors for college students pre-vaccine. In 2021, Massar et al. [20] studied evolving physical activity behaviors during reopening. In 2022, Mair et al. [21] created JitaBug, a personalized app for health intervention. Also, three studies [22,23,24] examined changes in sleep and activity during COVID-19 restrictions. Nevertheless, due to the disparities in objectives, methodologies, and outcomes, the task of synthesis remained unfeasible.

All studies used a commercialized physical activity tracker brand, placed at different points around the trunk, upper, and lower limbs. Less than half of the studies [18,21,22] reported their data collection sampling frequency, which ranged between 30 and 100 Hz, a suitable sampling rate frequency due to the low-pace nature of protocol tasks [34]. On the other hand, none of the studies calculated or reported test–retest reliability and minimum detectable change values of the sensors. Given the motion sensors’ susceptibility to test–retest variability, their reliability is compromised, leading to an elevated minimum detectable change. To address this specific issue, the authors recommend that future studies incorporate evaluations of device accuracy, which should encompass comprehensive measures of reliability. This approach is in line with the recommendation by Düking, P. et al. [35].

Regarding derived sedentary parameters, all studies [18,19,20,21,22,23,24] employed stepping activity-related calculations such as total steps per minute, per day, or over a specified study duration. Heart rate data [18,19,23,24] were collected and examined for variability and resting rate, while four studies [19,20,22,23,24] presented data related to sleep duration, bedtime, and time spent lying down. Even in the absence of uniform reporting standards for physical activity metrics, these parameters continued to play a crucial role in deciphering sedentary behaviors within the unique contexts of their research goals.

Among the seven studies, only the studies by Ong et al. and Leone et al. [22,24] distinctly highlighted their respective novelty in the state of the art. Study [22] introduced a novel platform for the automatic quantification of energy expenditure, and study [24] demonstrated the impact of COVID-19 restrictions on physical activity levels through innovative rest–activity rhythm and hierarchical clustering approaches.

The studies reviewed in this discussion section have several limitations that can be categorized into three main areas and should be considered when interpreting their findings.

(1) Sample selection: Several studies relied on convenience samples, which may not be representative of the wider population [20,23,24]. Additionally, some studies had skewed gender ratios [20] or overrepresented certain ethnic groups [19]. These limitations limit the generalizability of the findings to the wider population.
(2) Data collection methods: Some studies used consumer-grade wearables to measure physical activity and sleep [21,22,24]. While these devices are convenient and widely used, they may be less valid than research-grade devices for assessing activity intensity and sleep quality [21]. On the other hand, these devices present a multitude of advantages for health research. They are not only more cost-effective than premium research devices [36], but also boast comfort in wear [37], making them easily accessible to consumers at an affordable price [38]. Additionally, some studies lacked pre-lockdown data for comparison [20,23], which makes it difficult to determine the true impact of COVID-19 on physical activity and sleep.
(3) Data analysis: Some studies made assumptions about the linearity of relationships or the constancy of model parameters and were unable to use certain data analysis techniques due to the small sample size or noisy data [18]. These methodological limitations may have affected the accuracy of the findings.

Another interesting observation is that three of the studies [19,20,24] included in this summary rely on self-reported data to some extent. This raises concerns about the accuracy of the findings, as self-reports can be biased and unreliable. Future studies should explore ways to collect more objective data on physical activity using wearable devices or other technological solutions.

### 4.1. Future Directions

Due to the rapidly growing nature of wearable technology, we believe that wearable devices could be used in novel ways to assess related aspects of sedentary lifestyles to better prepare for the next world pandemic. Some potential applications include:

- Telehealth Integration: We now comprehend the negative repercussions of extended waiting periods on overall health during the COVID-19 lockdowns and the burden in post-pandemic times [39]. One way to uphold preventative medicine measures and potentially bridge this gap may be to leverage wearable devices. Wearable devices have the potential to seamlessly integrate with telehealth platforms, enabling healthcare practitioners to remotely monitor sedentary behavior. This integration would empower healthcare professionals to offer individualized recommendations and interventions aligned with individuals’ well-being, thereby amplifying the effectiveness of remote healthcare services. Simultaneously, these wearable devices possess the capability to meticulously trace prolonged sedentary patterns, facilitating the discernment of overarching trends and their associations with diverse health consequences. This valuable insight could significantly inform the creation of targeted interventions and strategies for fostering behavioral changes.
- Self-Psychological and Emotional Assessment: The past few years have highlighted the role of physical activity to alleviate psychosocial challenges during a lockdown [40]. Advanced wearables equipped with sensors can record objective physiological indicators such as heart rate variability and electrodermal activity, providing quantifiable insights into stress levels and emotional well-being [41]. In conjunction with other tools, such as a mood diary, or a self-reported emotional scale, wearables may offer another layer of self-awareness for people to assess emotional shifts in line with sedentary behavior and take preventative measures during lockdowns.

### 4.2. Limitations

It is important to note the lack of standardization in protocols, including variations in apparatus type, study duration, and different environments (such as controlled and day-to-day settings), poses challenges for cross-study comparisons. 

## 5. Conclusions 

Acknowledging the considerable influence of sedentary lifestyles, especially in the context of the worldwide health repercussions stemming from COVID-19 restrictions, numerous investigations have been initiated to assess these behaviors employing wearable sensors. Despite disparities in research methodologies, measured variables, and the absence of device accuracy assessments, the integration of wearable devices during the global pandemic offers a promising avenue for supplementary objective measurements and strategies to counter sedentary living. With rapid technological progress and the valuable insights gained from the recent pandemic, we strongly advocate for the widespread adoption and innovation of wearable technologies within the research and institutional sectors. These innovations can significantly bolster our better response to potential future global health crises.

## Figures and Tables

**Table 1:** Study characteristics.

Author [18,19,20,21,22,23,24]	Aim	Population Recruitment Characteristic	Selection Criteria	Participant Characteristics
Hojjatinia et al., 2022 [18]	To explore the effect of the COVID-19 pandemic on the dynamics of physical activity responses to digital message interventions.	Young adults	Inclusion Criteria: Ambulatory individuals aged 18–29 with no functional limitations, a visual impairment that hinders smartphone use, and fluency in English (both verbal and written). Participants must own a smartphone running iPhone iOS v10.0 or later or Android OS v7 or later and be willing to install the Random AIM and Fitbit apps. Exclusion Criteria: individuals engaging in 90 min or more of moderate or higher intensity physical activity per week, participating in mandatory physical activity programs, relying on mobility-assistive devices, diagnosed with cancer, cardiovascular disease, type I or type II diabetes, or metabolic syndrome, pregnant or planning to become pregnant in the next 6 months, or having any contradictions to physical activity based on the Physical Activity Readiness Questionnaire were excluded.	N: 28; Gender: M/F: 10/12 Mean age: 22.2 ± 1.7
Gilley et al., 2022 [19]	1. Evaluate self-reported physical, mental, and social health outcomes based on COVID-19 status. 2. Measure physical activity using consumer-grade wearable sensors (Fitbit). 3. Identify risk factors linked to COVID-19 positivity in a population of college students before the vaccine was available.	College students	Inclusion Criteria: Participants aged 18 years or older who are confirmed undergraduate or graduate students at the University of Michigan (whether on campus or at home). They must be capable of providing informed consent digitally, comfortable with reading and speaking English, and have access to the necessary resources for participating in an mHealth technology-based intervention (such as a smartphone or tablet device and internet access). Participants should also be willing to use their personal equipment or the Internet for the study. Exclusion Criteria: participants who did not complete the final (exit) assessment, did not complete a minimum of two monthly assessments, did not report a COVID-19 diagnosis, or did not wear the Fitbit device.	N: 1997; Gender: M/F/Other: 613/1367/16; Mean age: not reported (age ≥ 18)
Massar et al., 2021 [20]	To examine the changes in physical activity behaviors during the reopening phase, specifically exploring the potential influences of continued remote work and smartphone usage.	University students and staff	Inclusion: not reported Exclusion: not reported	N: 198; Gender: M/F: 61/137; Mean age: 26.1 ± 5.8;
Mair et al., 2022 [21]	This study aims to provide a comprehensive description of JitaBug, a personalized Just-in-Time Adaptive Intervention (JITAI) delivered through smartphones. The intervention is designed to support older adults in either increasing or maintaining their physical activity levels. Additionally, the feasibility of conducting an effective trial for the JitaBug intervention will be assessed, and the acceptability of JitaBug among older adults in a real-world setting will be explored.	Ambulatory, community-dwelling older adults who use a smartphone	Inclusion: ambulatory, community-dwelling older adults who use a smartphone Exclusion: knee injury participants excluded	N: 31; Gender: M/F: 14/17; Mean age not reported (Age: 56–72 years old)
Leone et al., 2022 [22]	In order to develop a platform that is widely accepted by users, two different sensor technologies were utilized to accurately measure and quantify the energy expenditure of older adults.	Aging Adults	Inclusion: not reported Exclusion: not reported	N: 11; Gender: M/F: 6/5; Mean age not reported (Age: 65–73 years old)
Capodilupo et al., 2020 [23]	The objective of this study is to measure and analyze changes in sleep/wake behavior, exercise behavior, and physiological markers of health during the period of physical distancing implemented during the COVID-19 pandemic.	WHOOP strap members (US-based)	Inclusion Criteria: Participants must have recorded their sleep for at least 120 out of the 135 days (89% of the days) between January 1 and March 9 in both 2019 and 2020. Additionally, participants should fall within the age range of 18 to 80 years on May 15th, 2020, which was the date when data were extracted for analysis. Exclusion Criteria: there are no specific exclusion criteria mentioned in the provided information.	N: 5436; Gender: M/F: 3900/1536 Mean Age: 40.25 ± 11.33
Ong et al., 2021 [24]	The aim of this study is to analyze sleep and physical activity (PA) data obtained from the “Health Insights Singapore” (hiSG) cohort.	Fitbit users from the Health Insights Singapore (high) study	Inclusion: young working adults between the age 21 and 40 Exclusion: not reported	N: 1824; Gender: M/F: 883/941; Mean age: 30.9 ± 4.6

**Table 2:** Study parameters and outcome measures.

Author [18,19,20,21,22,23,24]	Sensor Type and Specifications	Location on Body	Calculated Physical Activity Parameters	Sensor Assessment Protocol	Environment	Main Findings
Hojjatinia et al., 2022 [18]	ActiGraph—wGT3X-BT (Stage 1); 30 Hz sampling rate. Fitbit Versa/Versa Lite Smartwatch (Stage 2).	Waist (stage 1), on the participant’s dominant side at the midline of their thigh, wrist (stage 2)	(1) Stage 1: (1) daily step counts were measured as counts per minute, and (2) the mean overall speed of physical activity was calculated. (2) Stage 2: (1) Minute-level step counts and (2) heart rate were recorded. Before and after the pandemic declaration, seven features were extracted separately for weekends and weekdays. These features included initial delay, peak magnitude, peak delay, steady state, rise time, settling time, and effective time.	Stage 1 (Screening Stage): physical activity was measured over a period of 7 consecutive days in the field. Stage 2 (Intervention Stage): Participants who wore the device for at least 5 days with a minimum of 600 min per day and had an average of less than 21.4 min per day of moderate-to-vigorous physical activity (equivalent to 150 min per week) were invited to participate in the second stage of the study. They were asked to continue wearing the device for the next 6 months.	Day to day	Following the declaration of the pandemic, there was a significant decrease in daily step counts on weekdays (Cohen’s d = −1.40), while no significant change was observed on weekends (d = −0.26). The mean overall speed of the response related to physical activity (dominant pole magnitude) did not show significant changes on both weekdays (d = −0.18) and weekends (d = −0.21). However, there was limited consistency in the ranking of specific features of intervention responses before and after the pandemic declaration.
Gilley et al., 2022 [19]	Fitbit wristband	Wrist	(1) Number of steps per day, (2) heart rate, and (3) sleep	Participants were provided with Fitbit devices, which were sent to their homes via mail. They received instructions to wear the Fitbit continuously for a minimum of approximately 40 h per week during the 3-month monitoring period. The Fitbit was used to measure their physical activity, heart rate, and sleep patterns throughout this duration.	Day to day	A significant proportion of students (24% with moderate and 49% with severe anxiety levels) reported anxiety according to the State Trait Anxiety Index. About one-third of the students (33%) disclosed having a mental health disorder. Mental health issues were prevalent among the student population, and factors like substance use were linked to increased COVID-19 risk. These findings underscore the need to focus on innovative strategies that promote health and well-being and consider the long-term impacts of COVID-19 on college students.
Massar et al., 2021 [20]	Oura Ring;	Finger (not specified)	(1) Sleep, (2) physical activity (step count)	Participants were required to wear the Oura ring consistently throughout the entire duration of both the lockdown and non-lockdown periods, which lasted for a total of 8 weeks.	Day to day	After the lockdown, the reopening phase resulted in noticeable changes, including earlier sleep timing, increased physical activity, and changes in mental well-being. These changes were influenced by factors such as work/study arrangements and patterns of smartphone usage.
Mair et al., 2022 [21]	Fitbit (Charge 4); ActiGraph wGTX3-BT accelerometer at 100 Hz	Non-dominant wrist (both sensors)	(1) Daily steps, (2) activity minutes goal	Participants were given Fitbit activity trackers to wear on their nondominant wrist, with no other trackers nearby. They wore these devices continuously for 8 days, 24 h a day, except for bathing or showering. The accelerometers, set to collect data at 100 Hz, were synchronized with GMT and started recording at 6 AM the day after participants received them to ensure full data capture.	Day to day	The study indicates that a smartphone-delivered Just-in-Time Adaptive Intervention (JITAI) is a well-accepted method of supporting physical activity (PA) in older adults within the community. The intervention was found to be feasible overall; however, user feedback suggests that further technical refinements to the JitaBug app are needed to improve its usability, engagement, and user satisfaction before progressing to effectiveness trials.
Leone et al., 2022 [22]	Shimmer3; 50 Hz	Chest	Human postures: (1) Standing, (2) Sitting, (3) Bending, (4) Lying down, (5) Walking	Each participant underwent three data acquisition sessions, each consisting of specific protocols involving standing, sitting, bending, and lying tasks. Each task lasted between 30 and 90 s. The protocols included sequences of static postures and walking at various speeds. The goal was to assess the classification performance of each individual sensory node, even in challenging situations that could potentially affect the accuracy of the classification.	Controlled	The study results demonstrated the platform’s ability to accurately measure energy expenditure using different sensing technologies. The wearable sensor achieved an average accuracy of 93.8% in posture classification, while the ambient sensor achieved 93.3% accuracy in walking activity classification. Combining the data from both sensors resulted in an approximately 4% improvement. As a result, the estimated energy expenditure had a relative error of less than 3.2% for each participant, successfully classifying high-level information such as postures and walking activities. These findings support the proposed architecture of the platform in terms of hardware and software. Novelty: “implementation of a platform that provides a novel tool for the automatic quantification of Energy Expenditure (EE)”.
Capodilupo et al., 2020 [23]	WHOOP strap	Wrist	(1) Sleep opportunity duration, (2) Social jet lag, (3) Sleep opportunity offset, (4) Sleep duration, (5) Exercise frequency, (6) Exercise type, (7) Exercise intensity, (8) Resting heart rate, (9) Heart rate variability	To assess the immediate changes in health-related behavior, the study defined the period from 1 January 2020 to 9 March 2020 as the baseline period. The period from 10 March 2020 to 15 May 2020 was identified as the physical distancing period, during which restrictions were implemented.	Day to day	The findings indicate that individuals demonstrated improved health-related behaviors, such as increased exercise intensity and longer sleep duration, during the period of physical distancing restrictions. There were positive changes observed in cardiovascular indicators of health. However, it remains unclear whether these changes can be directly attributed to the behavior changes or if other factors were involved.
Ong et al., 2021 [24]	Fitbit Ionic	Wrist	(1) Bedtime, (2) Waketime, (3) Time in bed (TIB), (4) Total sleep time (TST), (5) Sleep efficiency, (6) Step counts, (7) Time spent in MVPA, and (8) Resting heart rate	The collected physical activity (PA) data included total daily steps, moderate-to-vigorous physical activity (MVPA) minutes, resting heart rate levels, and 15 min interval step counts. To ensure data accuracy, certain filtering criteria were applied, excluding days with insufficient Fitbit wear time or atypical activity levels. Atypical activity levels were defined by specific thresholds for daily steps and sedentary minutes. The PA data analysis involved an average of 1375 participants at each time point, with an average daily wear time of 18–19 h.	Day to day	During the initial phase of COVID-19 mobility restrictions, physical activity (PA) was found to be more significantly impacted compared to sleep. An evaluation using the RAR (Response Analysis and Reporting) technique revealed that there was a variation in individuals’ responses to the lockdown, which could potentially be linked to different outcomes if the resolution of COVID-19 continues for an extended period. This suggests that prolonged restrictions may have varying effects on individuals’ physical activity levels and overall well-being. Novelty: “demonstrate how heterogenous groups are affected by using novel rest-activity rhythm and hierarchical clustering approaches”.

**Table 3:** Quality assessment questions.

Question	Hojjatinia et al., 2022 [18]	Gilley et al., 2022 [19]	Massar et al., 2021 [20]	Mair et al., 2022 [21]	Leone et al., 2022 [22]	Capodilupo et al., 2020 [23]	Ong et al., 2021 [24]
**Q1.** Is the hypothesis/aim/objective of the study clearly described?	Y	Y	Y	Y	Y	Y	Y
**Q2.** Are the main outcomes clearly described in the Introduction or Methods?	Y	Y	Y	Y	Y	Y	Y
**Q3.** Are the characteristics of the participants clearly described (including age, sex, and status as healthy/injured/pathological)?	Y	N	N	N	N	Y	Y
**Q4.** Are the inclusion and exclusion criteria described and appropriate?	Y	N	N	N	N	N	N
**Q5.** Are the main findings of the study clearly described?	Y	Y	Y	Y	Y	Y	Y
**Q6.** Are estimates of the random variability in the data for the main outcomes provided?	Y	Y	Y	Y	Y	Y	Y
**Q7.** Have actual probability values been reported for the main outcomes?	Y	Y	Y	Y	Y	N	Y
**Q8.** Are the participants representative of the entire population from which they were recruited?	Y	Y	Y	Y	Y	Y	Y
**Q9.** Are the setting and conditions typical for the population represented by the participants?	Y	Y	Y	Y	Y	Y	Y
**Q10.** Are the statistical tests used to assess the main outcomes appropriate?	Y	Y	Y	Y	Y	Y	Y
**Q11.** Are the main outcome measures used accurate (valid and reliable)?	Y	Y	Y	Y	Y	Y	Y
**Q12.** Is a sample size justification, power description, or variance and effect estimates provided?	N	N	N	N	N	N	N

Note: Y = Yes, N = No.

**Table 4:** Summary of key study and SENSOR characteristics.

Study/Wearable Sensor Charectaristic	Hojjatinia et al., 2022 [18]	Gilley et al., 2022 [19]	Massar et al., 2021 [20]	Mair et al., 2022 [21]	Leone et al., 2022 [22]	Capodilupo et al., 2020 [23]	Ong et al., 2021 [24]
Main sedentary measured paramaters	Step count, heart rate	Step count, heart rate, sleep	Sleep, step count	Step count, personalized physical activity time goal	Standing, sitting, bending, lying down (sleep), walking (step)	Sleep, heart rate, physical activity (step)	Step count, sleep, heart rate
Day–day environment	+	+	+	+	-	+	+
Controlled environment	-	-	-	-	+	-	-
Number of sensors	2	1	1	2	1	1	1
Upper limb placement	+ (Fitbit)	+	+	+	-	+	+
Lower limb placement	+ (Act.Gr)	-	-	-	-	-	-
Trunk placement	-	-	-	-	+	-	-
Commercial device/s	+	+	+	+	+	+	+
Sampling frequency: <50 Hz	+	N.R	N.R	-	-	N.R	N.R
Sampling frequency: 50–100 Hz	-	N.R	N.R	+ (Act.Gr)	+	N.R	N.R
Weight: <50 g	N.R	N.R	N.R	N.R	N.R	N.R	N.R

+ = Yes; - = No; N.R = not reported.

## Data Availability

No data were generated.
